# Galli–Galli Disease: A Comprehensive Literature Review

**DOI:** 10.3390/dermatopathology11010008

**Published:** 2024-02-07

**Authors:** Andrea Michelerio, Antonio Greco, Dario Tomasini, Carlo Tomasini

**Affiliations:** 1 Department of Clinical-Surgical, Diagnostic and Pediatric Sciences, University of Pavia, 27100 Pavia, Italycarlofrancesco.tomasini@unipv.it (C.T.); 2 Dermatology Unit, Ospedale Cardinal Massaia, 14100 Asti, Italy; 3 Dermatology Unit, ASST Valle Olona, 21052 Busto Arsizio, Italy; 4 Dermatology Clinic, Fondazione IRCCS Policlinico San Matteo, 27100 Pavia, Italy

**Keywords:** hyperpigmentation, acantholysis, keratin-5, skin diseases, genetic, skin diseases, papulosquamous

## Abstract

Galli–Galli disease (GGD) is a rare genodermatosis that exhibits autosomal dominant inheritance with variable penetrance. GGD typically manifests with erythematous macules, papules, and reticulate hyperpigmentation in flexural areas. A distinct atypical variant exists, which features brown macules predominantly on the trunk, lower limbs, and extremities, with a notable absence of the hallmark reticulated hyperpigmentation in flexural areas. This review includes a detailed literature search and examines cases since GGD’s first description in 1982. It aims to synthesize the current knowledge on GGD, covering its etiology, clinical presentation, histopathology, diagnosis, and treatment. A significant aspect of this review is the exploration of the genetic, histopathological, and clinical parallels between GGD and Dowling-Degos disease (DDD), which is another rare autosomal dominant genodermatosis, particularly focusing on their shared mutations in the *KRT5* and *POGLUT1* genes. This supports the hypothesis that GGD and DDD may be different phenotypic expressions of the same pathological condition, although they have traditionally been recognized as separate entities, with suprabasal acantholysis being a distinctive feature of GGD. Lastly, this review discusses the existing treatment approaches, underscoring the absence of established guidelines and the limited effectiveness of various treatments.

## 1. Introduction

Galli–Galli Disease (GGD) is a rare and poorly understood genodermatosis that exhibits an autosomal dominant inheritance pattern with variable penetrance [1,2], although sporadic cases lacking family history have been described [1]. First described by Bardach, Gebhart, and Luger in 1982 [3], GGD was identified in two brothers and has since been classified as part of the reticulate pigmented disorders of the skin (RPDS) group [4].

Clinically, GGD is characterized by erythematous macules and papules with reticulate hyperpigmentation affecting the flexural areas, including the axillary, inguinal, and neck regions. During the inflammatory phase of the disease, itchy, erythematous, vesicular, and/or scaly papules may manifest in the same distribution, as well as on the trunk and proximal extremities [5,6,7]. Comedo-like lesions or pitted acneiform scars on the face may occasionally occur [6,8,9]. An emerging consensus posits a distinct GGD subset characterized by an atypical phenotype. While histopathological features remain consistent with classical GGD, this variant diverges in its presentation of brown macules on the trunk, lower limbs, and extremities, almost absent the hallmark reticulated hyperpigmentation in flexural areas [5].

The clinical phenotype of classic GGD bears a striking resemblance to Dowling-Degos disease (DDD), another rare autosomal dominant genodermatosis characterized by reticulate pigmentary macules of the flexures, comedo-like lesions on the back, neck, and face, and perioral pitted acneiform scars [10,11]. In addition, there have been various reports in the literature that include atypical presentations of DDD or additional uncommon findings [10]. This significant overlap between GGD and DDD has led to ongoing confusion and misdiagnosis [12]. Traditionally, the differentiation has been primarily histopathologic, with suprabasal acantholysis serving as a distinctive feature that is historically attributable to GGD, thereby categorizing it as an acantholytic variant of DDD [5]. Finally, molecular genetic analyses have identified mutations in the *KRT5* and *POGLUT1* genes in some cases of both GGD and DDD; therefore, GGD is now referred to as a variant of DDD [7].

The aim of this review is to provide a comprehensive synthesis of the existing knowledge on GGD, encompassing its etiology, clinical presentation, histopathology, diagnosis, and treatment options.

A further aim is to substantiate the hypothesis that GGD and DDD may, in fact, constitute two distinct clinical phenotypes of a unique pathological entity.

## 2. Literature Search

A search of the dermatologic literature was performed using the PubMed search engine to identify any reports of Galli–Galli disease since it was first described in 1982 to September 2022. The following key words and their synonyms were used singly or in combination [Galli–Galli Disease], [acantholytic], [acantholysis], and [Dowling-Degos disease]. The language was not limited to English. Various types of publications were considered for inclusion, such as original articles, reviews, case series and case reports, comment articles, and letters to editors. The reference lists of all articles, reviews, and case reports included were hand-searched to find additional eligible articles.

## 3. Pathogenesis

Despite the fact that GGD and DDD have very similar clinical aspects to each other, GGD has traditionally been considered to be a separate entity from DDD because suprabasal acantholysis is histopathologically observed only in GGD [12]. Advances in genetics have identified key mutations in genes such as *KRT5* and *POGLUT1* as pivotal in the pathogenesis of GGD. Intriguingly, these mutations have also been reported in cases of DDD [7,13].

### 3.1. KRT5

Keratin 5 (KRT5) protein coding gene, which was initially identified in DDD in 2006, is the main gene involved in the pathogenesis of GGD [8]. This gene is located on chromosome 12 (12q13.13), contains nine exons, and is predominantly expressed within the basal cells of the epidermis [14]. Its alteration, which is mainly caused by frameshift mutations, causes premature stop codons and results in haploinsufficiency (an insufficient production of the gene product) [5,14].

**Figure 1:**
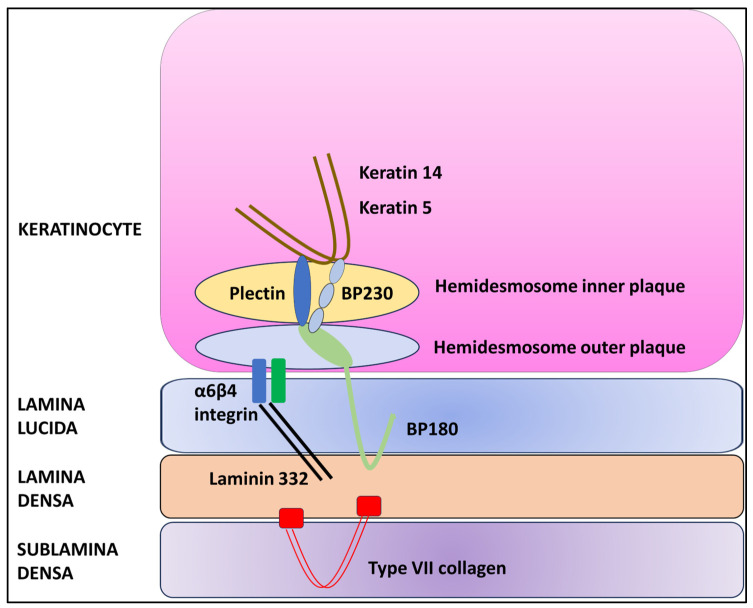
Schematic diagram of the dermal–epidermal junction.

KRT5 is a protein that forms intermediate filaments, or heterodimers, with cytokeratin 14 (KRT14) [14,15]. Both proteins play a critical role in attaching keratinocytes together and anchoring the epidermis to the dermis (Figure 1) [14,15,16].

In the presence of haploinsufficiency, the ratio of KRT5 to KRT14 is altered, resulting in a change in intermediate filament structure and impaired binding to desmosomes and hemidesmosomes and leading to the characteristic histopathologic alteration associated with GGD, i.e., acantholysis [7,14]. However, the precise pathogenetic mechanism that causes acantholysis is still unknown [7,17,18]. In addition, KRT5 plays a key role in epidermal differentiation and the uptake and degradation of melanosomes by keratinocytes [19]. In fact, mutations of the *KRT5* gene have been associated with hyperplasia of epidermal ridges and the accumulation of melanin, resulting in the typical reticular hyperpigmentation [14,19].

Mutations in the *KRT5* gene were identified in 21 out of 69 patients diagnosed with classical Galli–Galli disease [8,9,18,20,21,22,23,24]. The most common genetic alteration in the *KRT5* gene is the c.418dupA missense mutation, which was found in 13 of the 21 patients with a *KRT5* mutation [18,21,22,24]. Notably, this specific mutation has also been observed in DDD [5,7,18,21,22,24]. The c.418dupA mutation results in the insertion of an adenosine at position 418, causing a frameshift in the gene sequence [5,8].

Several de novo mutations have been identified in the *KRT5* gene over the years in patients with classical GGD, most of which have not been linked to distinct phenotypes [24] An Arabian patient harbored a substitution of thymine for cytosine at position 2 (c.T2C), resulting in the destruction of the NlaIII endonuclease recognition site and a change in the codon of coding mRNA, which resulted in haploinsufficiency [20].

An Asian-American woman was identified as carrying a c.38dupG mutation, which led to the insertion of a guanine at position 38 in exon 1, subsequently causing a premature stop codon and resulting in KRT5 haploinsufficiency [8].

A Caucasian patient had the c.14C > A mutation, which resulted in the substitution of cytosine for adenosine [21].

A Caucasian patient with a segmental phenotype was found to carry a c.476C4T mutation. This mutation caused a substitution of leucine for proline at position 159, resulting in the production of a nonfunctional protein [23].

An Indian family was found to carry a mutation c.10C > T, in which cysteine was replaced by threonine at position 10 [9]. In addition to the classic symptoms, this family exhibited hyperpigmentation on the extremities and face. Furthermore, multiple hypopigmented macules were symmetrically distributed over the trunk.

### 3.2. POGLUT1

With the development of whole-exome sequencing (WES), mutations in *POGLUT1* (encoding protein O-glucosyltransferase 1) have been shown to underlie some cases of GGD/DDD [13,25].

**Figure 2:**
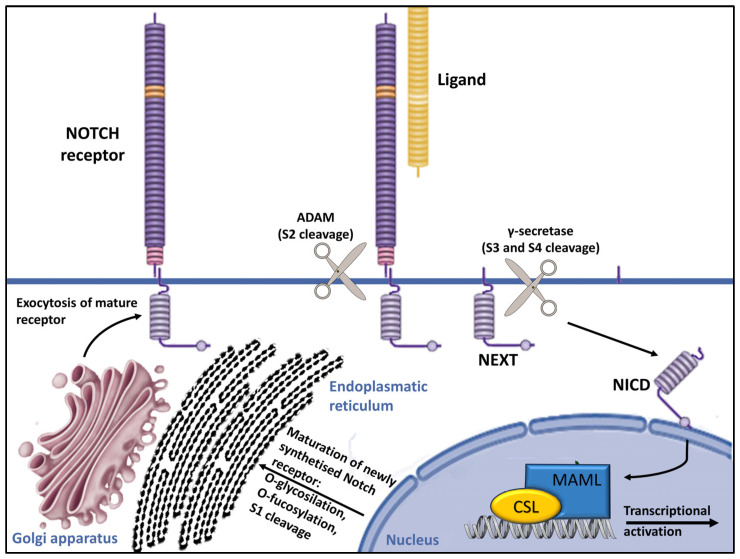
Overview of Notch signaling. Notch receptors undergo initial translation in the endoplasmic reticulum and are then trafficked to the Golgi apparatus. During trafficking, O-linked and N-linked glycosylations occur. Then, in the Golgi apparatus, Notch receptors are cleaved into heterodimers (S1 cleavage) and transported to the cell membrane. Upon reaching the cell surface, the receptor is activated through ligand binding. This ligand–receptor interaction triggers transendocytosis in the neighboring cell, inducing a conformational alteration in the Notch receptor and exposing the S2 site. This site is cleaved by ADAM metalloproteases (S2 cleavage), generating a membrane-anchored Notch extracellular truncation (NEXT). The S2 cleavage exposes S3 and S4 sites, facilitating further proteolytic cleavage by the γ-secretase complex (S3/S4 cleavage). This results in the liberation of the notch intracellular domain (NICD), which translocates to the nucleus. There, it binds to CBF1/RBP-Jκ/Su(H)/Lag-1 (CSL), which is known as RBP-Jκ in vertebrates. In its basal state, CSL functions as a transcriptional repressor by associating with corepressor (Co-R) proteins. NICD binding converts it into a transcriptional activator by forming a ternary complex with mastermind-like (MAML) proteins, thus initiating the transcription of downstream target genes [29].

The *POGLUT1* gene, which is located on chromosome 3q13.33, encodes a protein that plays a crucial role in posttranslational modifications within the Notch pathway, transferring glucose molecules to serine residues of epidermal growth factor-like (EGF-like) domains within the endoplasmic reticulum [13,26,27]. The addition of the O-glucose residues by POGLUT1 plays a crucial role in the folding of Notch pathway receptor proteins within the endoplasmic reticulum, which is essential for their transport to the cell surface (Figure 2) [26,27,28,29].

The Notch pathway comprises four receptors and five ligands, and it is responsible for regulating skin homeostasis. It affects the proliferation and differentiation of melanocytes and keratinocytes, as well as their interaction. The alteration of this pathway leads to keratinocyte differentiation abnormalities, as evidenced by the increased expression of KRT5 and KRT14 in immunohistochemistry, as well as disturbance in skin pigmentation, as demonstrated by the abnormal migration of melanoblasts and melanocytes to ectopic sites in animal models [13].

In 2014, Basmanav et al. discovered various mutations in the *POGLUT1* gene in individuals diagnosed with DDD who exhibited a disseminated pattern of brownish macular and lentiginous lesions on the extremities, trunk, and neck, rather than the typical domination of flexural folds commonly seen in classical DDD [13]. Notably, six of these cases had previously been classified as atypical GGD in an earlier study by one of the Authors [21]. They identified two nonsense mutations (c.11G > A and c.652C > T) and a splice-site mutation (c.798-2A > C) [13]. These mutations led to truncated proteins with compromised stability, subsequently reducing the glycosylation of receptor proteins in the Notch pathway [13].

The observed reduction in glycosylation has been demonstrated in animal models, revealing abnormalities in keratinocyte and melanocyte development and differentiation [13]. The *POGLUT1* gene significance in cell differentiation is further supported by immunohistochemical analysis, which shows increased expression in the upper layers (spinous and granular) and a decrease in expression by about 50% in affected individuals. This is consistent with heterozygous mutations leading to a loss of function [13,25,27,30].

Histologically, the skin lesions of individuals with *POGLUT1* mutations have showed a digitiform epidermal ridge hyperplasia with pronounced hyperpigmentation at the tips of the rete ridges, focal hypergranulosis, and, notably, some small horn cysts and minor acantholysis.

In recent years, three additional cases of GGD with *POGLUT1* mutation were identified: one with a c.1013delTinsAAC [30], one with c.652 > T [4], and one with c.798-2A > C [31].

### 3.3. A Possible Role of PSENEN?

The presenilin enhancer, gamma-secretase subunit (PENSEN) gene plays a key role in Notch signaling activation, providing instructions for a protein called gamma-secretase subunit PEN-2 (shortened to PEN-2) [32]. It is a transmembrane protease composed of four essential protein subunits (the γ-secretase complex) located in the cell membrane, where it cleaves many different transmembrane proteins. This cleavage is an important step in several chemical signaling pathways that transmit signals from outside the cell into the nucleus, including Notch signaling. (Figure 2) [29,32,33,34,35].

The *PSENEN* gene, which has been extensively studied in the context of neurodegeneration, has been linked to over 300 mutations primarily associated with early-onset Alzheimer’s disease [32]. Recently, mutations in *PSENEN* have also been discovered in patients with familial hidradenitis suppurativa (HS) [36]. These mutations result in presenilin dysfunction and impaired cleavage activity, particularly affecting substrates like Notch receptors [37,38].

A cooccurrence of HS and DDD was first mentioned in 1990 [39] and subsequently confirmed in numerous case reports [33]. Pavlovsky et al. described four more individuals with HS–DDD [33] Importantly, the study also identified a founder mutation in the *PSENEN* gene [33].

Del Mar et al. reported the first case of GGD associated with HS but did not conduct any genetic assays to identify mutations that had been previously reported in such conditions [34]. Although the precise pathogenic mechanism linking GGD and DDD with HS remains unclear, current evidence suggests that mutations in *PSENEN* may be involved, similarly to alterations in *POGLUT1*, affecting the Notch signaling pathway [33,38,40,41].

## 4. Clinical Presentation

To the best of our knowledge, 69 cases of GGD have been described in the literature, including 27 males and 42 females (female-to-male ratio of 1.5) with a mean age at diagnosis of 49 years (range 17–77 years). Difficulty in diagnosing may account for the low prevalence of the dermatosis [5]. In our literature review, a specific age at disease onset and time of diagnosis was provided in 52/69 cases, with an average delay of 15 years in diagnosis. Remarkably, in two cases, the delay exceeded 40 years [21,24]. It is noteworthy that in 15 cases the exact age of onset or diagnosis remained unspecified [30,34,42,43,44,45]. This underscores the challenges in either symptom recognition or the diagnostic process itself.

The disease involves individuals between the second and ninth decades of life, reaching its peak in the third and fifth decade [5,7,45]. The majority of patients were Caucasians (52/63) [1,2,3,4,6,18,19,21,22,23,24,31,42,43,45,46,47,48,49,50,51,52], followed by Indians (6) [9,44], Asians (5) [53], Africans (1) [34], Arabians (1) [20], Asian-Americans (1) [8], Japanese (1) [30], and Latin Americans (1) [54]. In one case the race is unspecified [5].

**Figure 3:**
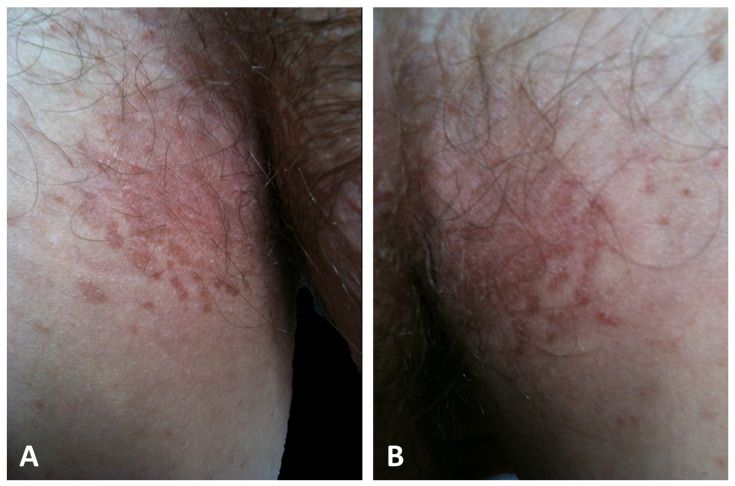
Typical presentation of GGD with hyperkeratotic papules and erythematous brownish macules that coalesce into patches with a reticulated appearance on the inguinal (**A**) and axillary folds (**B**).

It is increasingly evident that two distinct presentations of GGD exist, rather than a single phenotype, as formerly believed [5,21,49]. The first presentation, which is observed in the majority of patients (42/69) and consequently designated as typical, is characterized by progressive symmetrical, reticular, and partly lentiginous pigmentation of the flexures (the neck, axillary, mammary, and inguinal folds) in combination with erythematous brownish macules and papules, which sometimes coalesce into reticulated plaques (Figure 3) [1,2,3,6,8,9,18,20,21,22,24,34,42,46,47,48,49,52,53,54].

**Figure 4:**
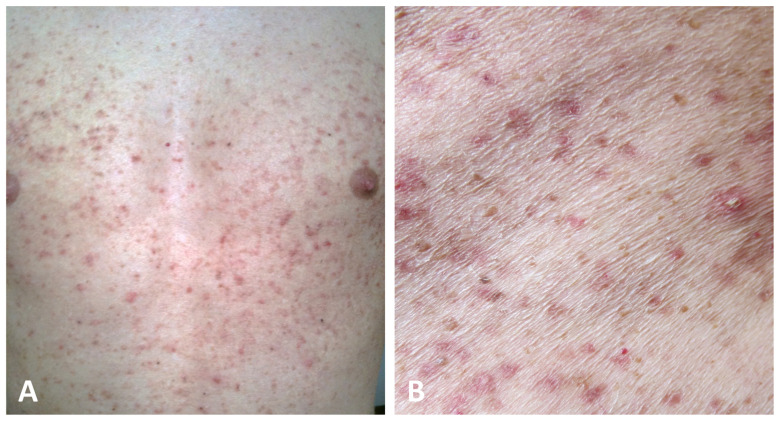
Atypical presentation of GGD with dissemination of brownish macules and erythematous papules on the trunk with sparing of the folds (**A**,**B**).

The second presentation, which has been identified in a minority of cases (25/69) and hence defined as atypical, is characterized by erythematous macules and papules widely distributed over the trunk, neck, back, abdomen, and limbs without the involvement of the flexural folds (Figure 4) [4,5,19,21,30,43,44,45,50,51,55,56,57].

One patient presented with brown macules in a segmental variation involving her left thigh due to a type-1 segmental inherited disorder resulting in a *KRT5* p.P159L mutation [23]. Because the patient exhibited a localized distribution without generalized involvement, it was postulated that this could represent a type 1 segmental manifestation resulting from a postzygotic mutation [58]. In contrast, type 2 mosaicism arises from postzygotic loss of heterozygosity, with loss of the wild-type allele leading to generalized involvement with one segment more affected than the rest of the skin. This event was not observed in this case, as the remaining skin was unaffected [23,58]. Only a single case could not be categorized as typical, atypical, or segmental due to insufficient data [31].

**Figure 5:**
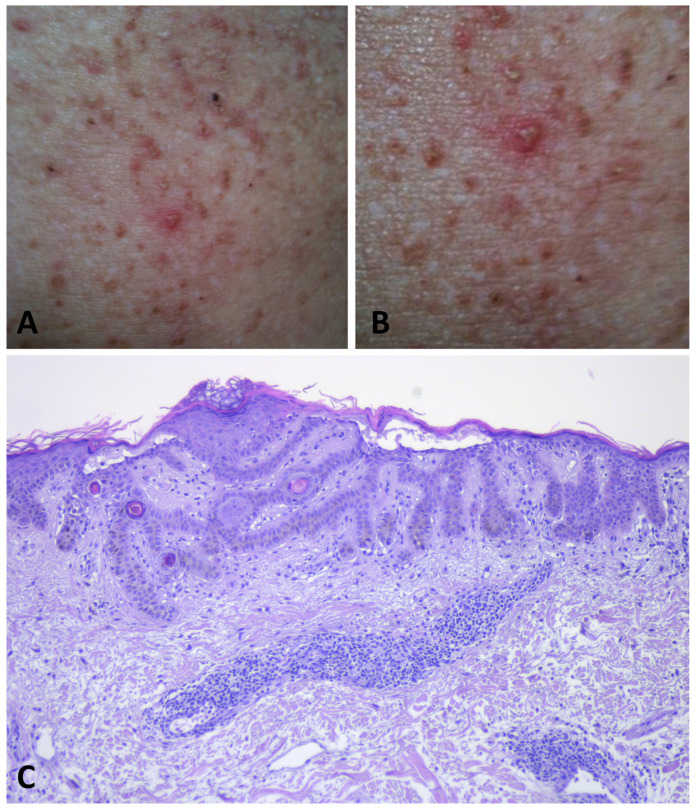
During flare phases of GGD, multiple vesicles develop, leading to intensely pruritic erosions and crusts on the skin (**A**,**B**). Histopathology shows a superficial dermal perivascular lymphocytic infiltrate. Apical acantholysis is apparent in the epidermis (**C**).

During the inflammatory phase, the maculo-papular lesions become more pronounced and develop into pruritic vesicles, erosions, and crusts (Figure 5) [5,49,54].

Various etiological factors may trigger the inflammatory response. In a study conducted by Hanneken et al. in 2011, sweat was identified as the primary aggravating factor in 10 of the 18 patients, followed by elevated temperature (9/18) and ultraviolet radiation (7/18), with these three triggers coexisting in four patients [21]. Only in a single case was decreased temperature the possible triggering factor. Moreover, exacerbation following dialysis treatment was first reported in the Literature [45].

In GGD, a notable correlation has been identified between genotype and phenotype. Specifically, mutations in the *KRT5* gene have been observed to cause cutaneous involvement primarily in the flexural areas, resulting in the typical clinical manifestation [21]. Among the 43 patients exhibiting the typical presentation, the mutation in the *KRT5* gene was tested in 23 individuals, of which 20 were found to have the mutation. The mutation was not tested in the remaining 20 individuals [8,9,18,20,21,22,24]. Interestingly, the sole reported case of segmental GGD, which has been attributed to the type 1 mosaicism phenomenon, was associated with the p.P159L type *KRT5* gene mutation [23]. In contrast, among the 24 patients with the atypical presentation, no mutation was detected within the *KRT5* gene [4,5,19,21,30,43,44,45,50,51,55,56,57]. These findings were corroborated by Basmanav et al., who identified mutations in the *POGLUT1* gene in individuals presenting with atypical GGD [13,21]. Similarly, studies conducted by Kono et al. [30] and Rundle et al. [4] revealed that patients with atypical variants were characterized by mutations in the *POGLUT1* gene. Unfortunately, data regarding *POGLUT1* mutations in the other cases of atypical GGD are not available [19,43,44,45,49,50,51,55,56,57]. Table 1 summarizes the clinical data of the patients.

## 5. Histopathology

Skin biopsy plays a crucial role in diagnosing GGD. Characteristic features consist of filiform, digitated hyperplasia of the epidermal ridges, basal hyperpigmentation of the tips of rete ridges, multiple foci of acantholysis in the upper spinous and/or granular layer, and narrowing of the suprapapillary epidermis with widened granular cell layer [5,7,8,49,50]. Some dyskeratotic keratinocytes may also be seen [3,4,8,30,31,43,45,50,51,55,56]. It should be noted that these findings are not consistently present and may vary depending on the stage of the disease.

During the inflammatory phase, an infiltrate of lymphocytes, histiocytes, and sparse eosinophils is observed in the papillary dermis [5,19,50]. Foci of epidermal spongiosis close to acantholytic foci may eventuate in papulovesicular and scale-crusted lesions (Figure 5C).

**Figure 6:**
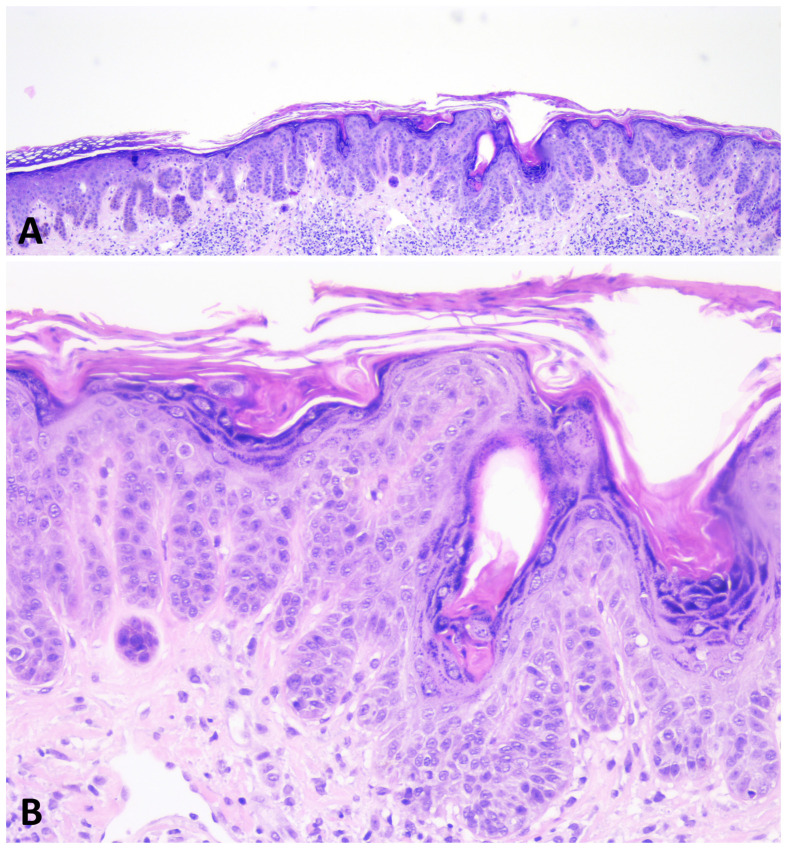
During the noninflammatory phase of GGD, the dermatosis is histologically indistinguishable from DDD. The epidermis shows thin, elongated, branching rete ridges with basal hyperpigmentation. There is mild hyperkeratosis overlying the epidermis (**A**,**B**).

During the noninflammatory phase, dermatosis runs an asymptomatic course and may be clinically and histologically indistinguishable from DDD. Histopathologically, acantholysis, if any, is minimal, and there is no evident dermal inflammation (Figure 6).

In this stage, only digitate hyperplasia of epidermal ridges and melanin deposits in the papillary dermis can be detected. Additional findings that may be observed include follicular comedo-like cysts, hyperkeratosis, dyskeratosis, parakeratosis, acanthosis, and hypergranulosis [5,19,44,50].

## 6. Diagnosis of Galli–Galli Disease

Currently, there is no specific laboratory test or special staining for GGD. Therefore, diagnosis is based on clinical–pathological correlation and, when possible, genetic analysis [18,49].

Because several conditions may mimic GGD clinically and histopathologically, with often overlapping features, including DDD, Grover disease (GD), and Darier’s disease (DD), a high index of suspicion is required.

DDD is a benign genodermatosis belonging to the group of reticular pigmented dermatoses with autosomal-dominant inheritance patterns. Typically, skin lesions manifest during adulthood as symmetrical macular or reticular hyperpigmentation, as well as noninflammatory reddish-brown papules and plaques on body folds, wrists, face, and chest [10,11]. Hyperkeratotic itchy papules, pitted acneiform scars, comedo-like lesions, hypopigmented maculae, and palmar depressions are possible additional skin manifestations [60,61]. The occasional itch observed in DDD, similarly to GGD, may be related to an increase in the number of mast cells [10].

Both GGD and DDD can manifest in a generalized form featuring multiple small, only partially confluent, reddish-brown papules and plaques, as well as disseminated lentiginous hyperpigmentation over the trunk and extremities. The intriguing report by Wu et al on a family with both flexural and disseminated disease involvement blurred the lines between these two conditions [53]. Remarkably, two biopsies from the same patient yielded different results: a biopsy from the axillary area was diagnosed as GGD, while a biopsy from the abdominal area was identified as DDD.

Differentiation between two entities relies on biopsy. While DDD usually lacks the suprabasal acantholysis observed in GGD [7,12,49,60,61], both conditions share similar features, such as epidermal ridge hyperplasia, hyperkeratosis, epidermal thinning of the epithelium, perivascular lymphocytic infiltrate, melanosis, and dermal fibrosis [12,53,60,61]. The need for repeated skin biopsies from different sites or serial sections to detect acantholysis has been emphasized [50]. Indeed, recent studies have highlighted that patients previously diagnosed with DDD showed foci of epidermal acantholysis on histopathologic sections from skin biopsies, which had been initially missed, relocating these cases within the spectrum of GGD [7]. This has led to the hypothesis that GGD and DDD are not two separate entities, but rather different clinical expressions of the same condition [7,9]. The same genetic background further corroborated this concept [7,24]. Recent genetic findings have revealed loss-of-function mutations in the *KRT5* gene in DDD cases [62], and mutations in the *POGLUT1* [13] gene have been identified in both DDD and GGD phenotypes.

GD is an acquired condition of unknown origin affecting mostly fair-skinned, elderly males [63] that is characterized by an intensely itchy eruption of discrete papules and vesicles on the trunk and proximal extremities [63,64,65]. The dermatosis is usually self-limiting, but it may persist for years with little response to therapy [63,66]. Clinically, GD may mimic the atypical variant of GGD, and the two conditions exhibit common triggers, such as heat and excessive sweating [63,67]. However, brown macules are not a typical feature of GD. The histopathologic hallmark of the GD is acantholysis, which is often associated with dyskeratosis and gives the lesions an appearance similar to Darier disease, Hailey–Hailey disease, or pemphigus. Spongiotic changes can be observed, as well [68]. In 2010, Fernández-Figueras et al. identified an additional pattern characterized by porokeratosis-like oblique columns of parakeratosis, lesions showing a nevoid or lentiginous silhouette, intraepidermal vesicular lesions, lichenoid changes with basal vacuolization and dyskeratosis, and dysmaturative foci with keratinocyte atypia [69]. Lentiginous elongation of rete ridges, however, is rarely observed in GD, and dyskeratosis is not a common finding in GGD [3,4,8,30,31,43,45,50,51,55,56].

The scientific literature has presented ambiguity concerning the classification of these specific dermatological conditions. In one prominent example, Ackerman provided commentary on a review by Gilchrist [1], wherein he advocated for the reclassification of cases diagnosed as GGD to be instead considered under the umbrella of GD [19,20,46,47,53] and cases of “extensive Grover’s-like eruption with lentiginous freckling” as GGD [43,70].

Moreover, while we have genetic studies recognizing specific mutations in the two forms of GGD, there is a notable absence of extensive genetic research on Grover’s disease. Recent evidence show the presence of *ATP2A2* mutations in Grover’s disease [71], while mutations in the *KRT5* gene, which are found in GGD, are rarely reported in GD [72]. This supports the idea that hereditary GGD is a distinct entity, while sporadic cases in adults may be part of the broader spectrum of GD.

Darier disease (DD) is a rare autosomal dominant genetic skin condition caused by mutations in the *ATP2A2* gene, which encodes SERCA2, a calcium ion transporter within the endoplasmic reticulum [59,73]. These mutations lead to altered desmosomal function with suprabasal acantholysis and dyskeratotic cells [59,73,74]. The dermatosis typically develops during puberty [73,74,75]. Clinical manifestations and disease severity can vary greatly, even within families with the same mutation [73,75,76]. Clinically, DD is characterized by warty plaques formed by coalescing firm, greasy, skin-colored, hyperkeratotic papules and primarily affects the seborrheic areas of the trunk and face [73]. Itching is common, occurring in 80% of patients. The involvement of flexures can lead to hypertrophic, fissured, and malodorous lesions, impacting the patient’s quality of life [73,77]. Triggers are similar to those in Hailey–Hailey disease, including heat, ultraviolet rays, sweat, infection, and mechanical rubbing [73,75]. Other clinical features observed in DD patients include short stature, subungual and palpebral hyperkeratosis, palmoplantar punctiform depressions, leukonychia, and longitudinal erythronychia with nail fragility. DD can also affect the oral cavity, presenting with symptoms such as macroglossia, gingival hyperplasia, leukokeratosis, and cobblestone-like papules [73,75,76]. Histologically, DD is characterized by suprabasal acantholysis and dyskeratotic keratinocytes (corp ronds) in the stratum spinosum and granulosum [73,75,76]. DD patients may experience comorbidities, such as neuropsychiatric disorders, fungal and bacterial infections, and potentially fatal herpes simplex virus reactivation [73,75,76].

## 7. Dermoscopy

Dermoscopy and reflectance confocal microscopy (RCM) are not usually used for the diagnosis of GGD; however dermoscopy and confocal findings have been described in a 48-year-old woman [56]. Dermoscopy revealed distinct features: one of the hyperkeratotic papules exhibited a central brown, mottled area surrounded by a whitish halo, while a lentigo-like macule displayed a peripheral pseudoreticular pattern with a central brown homogenous area.

To further investigate the condition, RCM imaging was performed [56]. RCM examination of the hyperkeratotic papule showed focal dark clefts with multiple bright, roundish cells at the epidermal level. RCM imaging of the lentigo-like macule revealed multiple, branched, deer antler-like highly refractile structures at the dermal–epidermal junction.

Epidermal dark clefts observed through RCM corresponded to suprabasal acantholysis, while round bright cells indicated dyskeratotic keratinocytes. Additionally, the characteristic elongated rete ridges observed in RCM correlated well with the bright, deer antler-like structures seen in confocal images and the lentigo-like dermoscopic presentation.

## 8. Therapy

Currently, there is a lack of established guidelines for the treatment of Galli–Galli disease (GGD), and limited evidence exists supporting the effectiveness of any specific treatment options [5,45].

However, certain measures can be recommended to patients, such as avoiding tight clothing that may cause irritation and sun protection to prevent the exacerbation of lesions [45].

Some therapeutic approaches, including vitamin A derivatives and laser therapy, have shown some efficacy [4,22]. Other conventional treatments, such as corticosteroids, emollients, calcineurin inhibitors, antihistamines, phototherapy, and antibiotics, have demonstrated limited efficacy [1,5,55].

Topical corticosteroids: Topical corticosteroids have generally been ineffective in most GGD cases, providing only occasional relief from itching and burning without significant clinical or histopathological improvement [19,31,57,78]. In some instances, the use of topical steroids has even resulted in worsened symptoms [6,34]. However, they are commonly used as an initial treatment during diagnostic investigations and in the early stages of the disease [1,5,19]. Commonly used topical steroids include clobetasol 17-propionate, betamethasone, and triamcinolone cream 0.1% [1,2,4,6,51]. Potential side effects of topical steroids include skin atrophy, telangiectasias, folliculitis, hypertrichosis, striae distensae cutis, and erythema.

Calcineurin inhibitors: Calcineurin inhibitors, such as tacrolimus and pimecrolimus, work at the molecular level by inhibiting cytokine transcription and T lymphocyte activation, thereby regulating the immune response [79,80]. Although there is only one reported case of initial improvement in GGD lesions with off-label use of these inhibitors, subsequent discontinuation was necessary due to cutaneous Candida spp. infections [49]. The side effects of calcineurin inhibitors can include folliculitis, furuncles, dermatitis, burning, irritation, itching, and intertrigo [80].

UVB phototherapy: UVB phototherapy, which utilizes ultraviolet radiation, has rarely been used in GGD due to its limited benefits and extensive side effects [44]. While it may provide some relief from itching, adverse reactions can include erythema, blistering, hyperpigmentation, skin photoaging, increased neoplastic risk, and even the development or exacerbation of GGD [2,19,44].

Laser therapy: Laser therapy, including the Er:YAG laser, pulsed light (IPL), and electrofluorescence, has shown equal efficacy in treating GGD by removing pathological tissue and promoting the regeneration of new epidermal tissue [22,52]. The Er:YAG laser is advantageous due to faster lesion resolution and fewer side effects, such as atrophic scarring and pigmentation changes [52]. An interesting case report from 2011 describes a 68-year-old patient who had chronic itching for 5 years, was diagnosed with GGD, and was treated with the Er:Yag laser at the axillary level in two successive sessions. After anesthesia with prilocaine, a laser with a spot size of 5 mm, a frequency of 8 Hertz, and a pulsed energy of 1200 millijoules was used with excellent results [22]. However, limited availability, high maintenance costs, and potential scarring and dyschromic outcomes in cases with extensive lesions may outweigh the benefits of laser therapy. Nevertheless, it currently represents the best local treatment option for GGD [10,22].

Systemic corticosteroids: Systemic corticosteroids, such as prednisone, have variable effects in GGD, ranging from partial resolution to worsening of lesions, and are typically used during disease flares [1,4]. Common side effects of systemic corticosteroids include hypertension, hyperglycemia, hypokalemia, osteoporosis, capillary fragility, cataracts, and psychiatric changes [81].

Vitamin A derivatives: Vitamin A derivatives, specifically retinoids like isotretinoin, alitretinoin, and acitretin, have shown promise as systemic treatments for GGD [5,82]. These retinoids regulate keratinocyte proliferation and differentiation by binding to the nuclear receptors all-trans retinoic acid receptor (RAR) and all-trans retinoid x receptor (RXR) [82]. Isotretinoin has demonstrated therapeutic effectiveness in treating GGD, with notable improvements observed in itching, rash resolution, reduced extent of rash, and improved erythema after just one month of treatment [51]. Similarly, alitretinoin has shown positive therapeutic outcomes, leading to decreased erosions, resolution of hyperpigmentation, and complete clinical response [6]. However, retinoids have numerous side effects, including facial erythema, pruritus, dermatitis, xerosis, joint pain, headache, fatigue, teratogenic effects, and potential psychiatric symptoms, such as anxiety, depression, and suicidal ideation [82,83].

Antibiotics: Antibiotics, particularly macrolides such as azithromycin, clarithromycin, and erythromycin, have rarely been used in GGD and are primarily employed in the treatment of reticulated pigmentary disorders of the skin [5,78].

Future therapies: Because steroids and retinoids are the first-line treatments for both GGD and Grover’s disease, it has been hypothesized that GGD might benefit from therapies that cause lesion regression in Grover’s disease, such as naltrexone and dupilumab. Naltrexone, a long-acting opioid receptor antagonist used to treat opioid and alcohol dependence [84], is utilized in dermatology at lower dosages (1–5 mg) for conditions often resistant to conventional treatments, e.g., psoriasis guttata, lichen planopilaris, HHD, and GD [84,85,86]. Low-dose naltrexone (LDN) has a shorter binding time and leads to a paradoxical increase in both ligands and receptors, resulting in pain relief and anti-inflammatory action [85]. It may also reduce proinflammatory cytokines and nitric oxide by binding to nonopioid receptors [73,84,85,86].

Dupilumab, a recombinant human IgG4 monoclonal antibody, inhibits the signal transduction of IL-4 and IL-13, which are cytokines involved in Th2-mediated inflammatory diseases [87]. Its off-label use has proven effective for Grover’s disease, which has a Th2-type inflammatory profile [88]. Using dupilumab as monotherapy at a loading dose of 600 mg for the first week, followed by a dose of 300 mg for each week to come for a total of 4 months, led to complete resolution of skin lesions and itching in three patients, suggesting the potential for treating acantholytic dermatoses like GGD [88]. Further research is needed to explore the efficacy of these emerging therapies for GGD and establish more definitive treatment guidelines.

## 9. Conclusions

GGD remains an intriguing and complex dermatological condition. While it manifests as an inflammatory acantholytic variant of Dowling-Degos disease (DDD), the two share significant clinical attributes, often leading to diagnostic overlaps. The genetic background of GGD is crucial, both for accurate diagnosis and understanding pathogenesis. Given recent advancements, the term “disseminated” is proposed as a more accurate descriptor than “atypical” for GGD phenotypes with diffuse involvement. More broadly, given the numerous overlaps, a proposal to combine GGD and DDD into a single disease entity deserves consideration.

## Figures and Tables

**Table 1:** Clinical characteristics of the patient with Galli–Galli disease in the literature.

Author	Gender	Ethnicity	Onset Age	Age at Diagnosis	Clinical Presentation	Distribution	Family History	Itch	Triggering Factors	Treatment	Genetic Analysis
Bardach, et al (1982) [3]	M	Caucasian	19	32	Erythematous maculo-papules with reticular hyperpigmentation	flexural	y (younger brother)	y	Heat	N.S.	N.P.
	M	Caucasian	15	41	Erythematous maculo-papules with reticular hyperpigmentation	flexural	y (older brother)	y	Heat, more frequent in summer	N.S.	N.P.
Mittag, et al (1986) [42]	M	Caucasian	N.S.	56	Erythematous maculo-papules with reticular hyperpigmentation	flexural	y	y	Heat	N.S.	N.P.
	M	Caucasian	N.S.	51	Erythematous maculo-papules with reticular hyperpigmentation	flexural	y	y	N.S.	N.S.	N.P.
	F	Caucasian	Puberty	46	Brown macules	flexural	y	y	Heat	N.S.	N.P.
	M	Caucasian	15	17	Brown macules	flexural	y	y	N.S.	N.S.	N.P.
Rütten and Strauß (1995) [46]	M	Caucasian	24	54	Erythematous maculo-papules with reticular hyperpigmentation, comedo-like lesions on face	flexural	no	y	Heat	SC: no improvement; Acitretin 30 mg/day: slight improvement	N.P.
De Deene and Schulze (1996) [47]	F	Caucasian	30	59	Erythematous maculo-papules with reticular hyperpigmentation	flexural	no	y	N.S.	TC/ATB: no improvement	N.P.
Braun-Falco, Volgger et al. (2001) [2]	M	Caucasian	49	53	Erythematous maculo-papules with reticular hyperpigmentation	flexural	no	y	Tretinoin 0.03%/urea 12%	UVB phototherapy/heliotherapy: slight improvement of itching	N.P.
Dhitavat et al. (2004) [59]	F	Caucasian	N.S.	42	Brown macules and erythematous papules	disseminated	no	y	Heat/UV/Sweat	Emollients: improvement of itching	*ATP2a2* unmutated/N.P. for *KRT5*
	F	Caucasian	29	39	Brown macules and erythematous papules	disseminated	no	y	Heat/UV/Sweat	Topical retinoids: no improvement	*ATP2a2* unmutated/N.P. for *KRT5*
El Shabrawi-Caelen et al. (2007) [19]	F	Caucasian	55	65	Brown macules and erythematous papules	disseminated	no	y	Heat/UV/Sweat	TC/Retinoids: no improvement	N.P.
	F	Caucasian	54	67	Brown macules and erythematous papules	disseminated	no	y	N.S.	TC/Retinoids/SC/UVB phototherapy: partial resolution	N.P.
Wu YH et al. (2007) [53]	F	Asian	8	30	Numerous symmetrically distributed hypopigmented or erythematous macules and papules on the chest, abdomen, axilla, posterior limbs, perioral, comedo-like lesions in the perioral area	disseminated	y		N.S.	N.S.	*KRT5* gene mutation not found
	M	Asian	childhood	55	Reticulated hyperpigmented macules and papules on his forehead, perioral area, chest, neck, elbow, and the back aspects of his forearm, axilla, hands, and feet, comedo-like lesions in the perioral area	flexural	y (father of the previous patient)		N.S.	N.S.	*KRT5* gene mutation not found
	M	Asian	8	28	Numerous symmetrically distributed hypopigmented or erythematous papules on the chest, abdomen, back, back aspects of the limbs, axilla, and perioral areas	disseminated	y (brother of the first patient)		N.S.	N.S.	*KRT5* gene mutation not found
	F	Asian	NA	50	Reticulated hyperpigmented macules and papules on his forehead, perioral area, chest, neck, elbow, and the back aspects of his forearm, axilla, hands, and feet, perioral	flexural	y (aunt of the first patient)		N.S.	N.S.	*KRT5* gene mutation not found
	F	Asian	NA	26	Reticulated hyperpigmented macules and papules on his forehead, perioral area, chest, neck, elbow, and the back aspects of his forearm, axilla, hands, and feet, perioral	flexural	y (cousin of the first patient)		N.S.	N.S.	*KRT5* gene mutation not found
Sprecher et al. (2007) [20]	F	Arabian	42	43	Erythematous maculo-papules with reticular hyperpigmentation	flexural	y (father and sister)	y	N.S.	N.S.	*KRT5* c.T2C
Gilchris T et al. (2008) [1]	M	Caucasian	36	41	Erythematous maculo-papules on neck, chest, back, proximal extremities with reticular hyperpigmentation in flexures	disseminated	no	y	Heat, summer	TC/SC/cyclosporin: improvement of itching but not of the lesions	N.P.
Müller CS et al. (2009) [48]	M	Caucasian	40	41	Erythematous maculo-papules with reticular hyperpigmentation	flexural	no	no	N.S.	N.S.	N.P.
	M	Caucasian	50	52	Erythematous maculo-papules with reticular hyperpigmentation	flexural	y (father)	y	N.S.	TC: no improvement	N.P.
	M	Caucasian	13	25	Brown macules and erythematous papules, comedo-like lesions in the perioral area	flexural	y (mother and maternal grandmother)	y	N.S.	TC/SC: no improvement	N.P.
	F	Caucasian	20	48	Brown macules and erythematous papules	flexural	y	no	N.S.	N.S.	N.P.
García-Salces et al. (2009) [54]	F	Latin American	27	29	Erythematous maculo-papules with reticular hyperpigmentation	flexural	no	y	Summer	N.S.	N.P.
Mota et al. (2010) [49]	F	Caucasian	20	55	Erythematous maculo-papules with reticular hyperpigmentation	flexural	y	y	Heat and sweating	Topical calcineurin inhibitors: effective at the beginning	N.P.
	F	Caucasian	56	58	Brown macules and erythematous papules	disseminated	no	no	N.S.	TC: no improvement	N.P.
Gomes et al. (2011) [55]	F	Caucasian	52	67	Brown macules and erythematous papules	disseminated	no	y	N.S.	Acitretin 25 mg/day and TC: slight temporary improvement	N.P.
Hanneken et al. (2011) [21]	M	Caucasian	41	42	Erythematous maculo-papules with reticular hyperpigmentation	flexural	y (father)	y	Sweat	N.S.	*KRT5* c.418dupA
	M	Caucasian	42	45	Erythematous maculo-papules with reticular hyperpigmentation	flexural	y (mother and sister)	y	Sweat/Mechanical stimuli	N.S.	*KRT5* c.418dupA
	F	Caucasian	32	47	Erythematous maculo-papules with reticular hyperpigmentation	flexural	y (six relatives)	y	Heat/UV/Sweat/Mechanical stimuli.	N.S.	*KRT5* c.418dupA
	F	Caucasian	21	52	Erythematous maculo-papules with reticular hyperpigmentation	flexural	y (relative of the third case)	y	Heat/UV/Sweat/Mechanical stimuli.	N.S.	*KRT5* c.418dupA
	M	Caucasian	15	25	Erythematous maculo-papules with reticular hyperpigmentation	flexural	y (relative of the third case)	no	No factor	N.S.	*KRT5* c.418dupA
	F	Caucasian	N.S.	47	Erythematous maculo-papules with reticular hyperpigmentation	flexural	y (relative of the third case)	y	No factor	N.S.	*KRT5* c.418dupA
	F	Caucasian	48	65	Brown macules	disseminated	y (sister)	y	UV	N.S.	*POGLUT1*
	F	Caucasian	42	47	Brown macules	disseminated	n	no	No factor	N.S.	*POGLUT1*
	M	Caucasian	18	61	Brown macules	disseminated	y (father)	y	Heat/UV/Sweat/Mechanical stimuli.	N.S.	*POGLUT1*
	F	Caucasian	53	63	Brown macules	disseminated	no	y	UV	N.S.	*POGLUT1*
	F	Caucasian	30–40	55	Brown macules	disseminated	no	no	No factor	N.S.	*POGLUT1*
	F	Caucasian	N.S.	52	Erythematous maculo-papules with reticular hyperpigmentation	flexural	no	y	No factor	N.S.	N.P.
	M	Caucasian	36	46	Erythematous maculo-papules with reticular hyperpigmentation	flexural	no	y	Heat/UV/Sweat/Mechanical stimuli.	N.S.	*KRT5* c.418dupA
	F	Caucasian	22	50	Erythematous maculo-papules with reticular hyperpigmentation	flexural	y (father)	y	Heat	N.S.	*KRT5* c.418dupA
	M	Caucasian	24	54	Erythematous maculo-papules with reticular hyperpigmentation	flexural	no	y	Heat/UV/Sweat	N.S.	*KRT5* c.418dupA
	F	Caucasian	48	58	Brown macules	disseminated	no	y	Heat/UV/Sweat	N.S.	*POGLUT1*
	M	Caucasian	49	62	Erythematous maculo-papules with reticular hyperpigmentation	flexural	no	y	Heat/Sweat	N.S.	*KRT5* c.418dupA
	F	Caucasian	N.S.	69	Erythematous maculo-papules with reticular hyperpigmentation	flexural	no	y	Heat/Sweat	N.S.	*KRT5* c.14C > A
Rongioletti et al. (2011) [50]	M	Caucasian	52	53	Erythematous maculo-papules with reticular hyperpigmentation	disseminated	no	y	N.S.	TC/ATB topicals/Antihistamines: lesion resolution	N.P.
Voth et al. (2011) [22]	M	Caucasian	63	68	Erythematous maculo-papules with reticular hyperpigmentation	flexural	no	y	N.S.	Yag Las er Ablative: lesion resolution	*KRT5* c.418dupA
Arnold et al. (2013) [23]	F	Caucasian	40	44	Brown erythematous macules with fine scales	segmental	no	no	N.S.	N.S.	*KRT5* c.476C4T
Schmieder et al. (2012) [24]	F	Caucasian	20	62	Erythematous maculo-papules with reticular hyperpigmentation	flexural	y (mother and maternal grandmother)	y	Winter climate	N.S.	*KRT5* c.418dupA
Reisenauer et al. (2014) [8]	F	Asioamericana	28	48	Erythematous maculo-papules with reticular hyperpigmentation, scattered hypopigmented macules, comedo-like lesions and pitted scarring on her face	flexural	no	y	Heat/Sweat/Tretinoin Cream	TS: no improvement; Topical retinoids: worsening	*KRT5* c.38dupG
Verma et al. (2014) [9]	F	Indian	15	57	Erythematous maculo-papules and reticular hyperpigmentation in flexural areas, hyperpigmentation on extremities and face, and hypopigmented macules	flexural	y (son, daughter and granddaughter)	y	Heat/UV/Sweat	N.S.	*KRT5* c.C10T
	M	Indian	15	32	Erythematous maculo-papules and reticular hyperpigmentation in flexural areas, hyperpigmentation on extremities and face, and hypopigmented macules	flexural	y (he is the son of the first patient)	N.S.	N.S.	N.S.	*KRT5* c.C10T
	F	Indian	15	39	Erythematous maculo-papules and reticular hyperpigmentation in flexural areas, hyperpigmentation on extremities and face, and hypopigmented macules	flexural	y (She is the daughter of the first patient)	y	Heat/UV/Sweat	N.S.	*KRT5* c.C10T
	F	Indian	15	19	Erythematous maculo-papules and reticular hyperpigmentation in flexural areas, hyperpigmentation on extremities and face, and hypopigmented macules	flexural	y (She is the granddaughter of the first patient)	N.S.	N.S.	N.S.	*KRT5* c.C10T
Desai et al. (2016) [44]	F	Indian	Puberty	55	Erythematous maculo-papules with reticular hyperpigmentation	disseminated	y	y	Heat/Humidity	N.S.	N.P.
	F	Indian	adolescence	35	asymptomatic, hyperpigmented macules localized to the face, neck and upper arms	disseminated					N.P.
Coelho de Sousa V et al. (2017) [56]	F	Caucasian	44	48	Erythematous maculo-papules with reticular hyperpigmentation	disseminated	no	y	N.S.	N.S.	N.P.
Dupuy et al. (2018) [51]	F	Caucasian	43	58	Erythematous maculo-papules with reticular hyperpigmentation	disseminated	y (mother)	y	Heat	Acitretin: discontinued for side effects; Isotretinoin: improvement	N.P.
Paolino et al. (2017) [57]	M	Caucasian	59	65	Erythematous maculo-papules with reticular hyperpigmentation and lentigines	disseminated	no	N.S.	N.S.	TC/SC/Retinoids: ineffective Acitretin 25 mg/day: slight benefit	N.P.
Seitz et al. (2018) [31]	F	Caucasian	27	37	Erythematous macules and papules	cannot be determined	N.S.	y	UV/Sweat	SC/TS: no benefit; Er:Yag Laser: lesion resolution	*POGLUT1* c.798-2A > C
Lőrincz et al. (2018) [18]	M	Caucasian	44	74	Erythematous maculo-papules with reticular hyperpigmentation	flexural	y (father, grandfather and two brothers)	N.S.	N.S.	Acitretin: improvement	*KRT5* c.418dupA
Kono et al. (2019) [30]	F	Japanese	End of third decade	50	Brown macules	disseminated	y (mother)	y	N.S.	N.S.	*POGLUT1* c.1013delTinsAAC
Yang A et al. (2019) [5]	M	N.S.	85	N.S.	Brown macules and erythematous papules	disseminated	no	y	Heat/UV/Sweat	TC/UVB: no improvement; Isotretinoin: slight improvement	*KRT5* gene mutation not found
Deenen et al. (2019) [6]	M	Caucasian	52	62	Brown macules with reticular hyperpigmentation	flexural	N.S.	y	N.S.	TC/SC: no improvement; Alitretinoin 30mg/day: effective	N.P.
Venning et al. (2019) [52]	F	Caucasian	20	40	Erythematous macules and papules	flexural	no	y	N.S.	Er:YAG Laser: lesion resolution	N.P.
Rundle et al. (2020) [4]	F	Caucasian	36	75	Erythematous maculo-papules with reticular hyperpigmentation	disseminated	y (son)	y	N.S.	TC/SC: no improvement; Acitretin 25mg/day: improvement	*POGLUT1* c.652 > T
Del Mar M et al. (2020) [34]	F	African	N.S.	33	Hyperpigmented macerated plaques (overlap with hidradenitis suppurativa)	flexural	N.S.	y	Triamcinolone 0.1%	Er:Yag laser: modest improvement	N.P.
Joshi et al. (2021) [45]	F	Caucasian	N.S.	77	Erythematous maculo-papules with reticular hyperpigmentation	disseminated	no	y	Heat/UV/Sweat/dialysis	SC: Slight improvement in itching. Patient refuses retinoids for side effects.	N.P.

y: yes; N.S.: not specified; N.P.: not performed. SC: systemic corticosteroids; TC: topical corticosteroids; ATB: antibiotics.

## Data Availability

The data presented in this study are available in the literature.
